# Ebselen: A Multifaceted Organoselenium Compound With Therapeutic Potential in Liver Diseases

**DOI:** 10.1155/omcl/9370771

**Published:** 2026-08-30

**Authors:** Lujain Bader Eddin, Shreesh Ojha

**Affiliations:** ^1^ Department of Pharmacology and Therapeutics, College of Medicine and Health Sciences, United Arab Emirates University, P.O. Box 15551, Al Ain, UAE, uaeu.ac.ae

## Abstract

Both oxidative stress and inflammation are considered the driving mechanisms in most liver pathologies. Therefore, identifying pharmacological compounds with antioxidant and anti‐inflammatory properties is central to curbing hepatic diseases. Ebselen is a synthetic organoselenium compound with glutathione peroxidase (GPx)‐like activity, demonstrating potent antioxidant and anti‐inflammatory effects experimentally. Ebselen targets several molecular mechanisms involved in hepatic cellular injury. It maintains redox‐sensitive signaling, neutralizes reactive free radicals, and regulates thiol‐dependent enzymes in experimental models of drug‐induced, ischemia‐reperfusion, and metabolic liver diseases. Studies provided experimental evidence for Ebselen’s role in suppressing lipid peroxidation, preserving mitochondrial dynamics, inhibiting inflammatory response, and protecting hepatocytes in cell lines as well as murine models of different liver injuries. Findings also reported an indirect effect on cellular demise and fibrogenesis through inhibiting the activation of hepatic stellate cells (HSCs) and decreasing collagen deposition. The hepatoprotective effects of Ebselen also extend to include reducing hepatic lipid accumulation and preventing steatosis induction in rodent models of liver fibrosis. This review summarizes the current available evidence on the hepatoprotective effects of Ebselen.

## 1. Introduction

Ebselen (2‐phenyl‐1,2‐benzisoselenazol‐3(2H)one), also known as SPI‐1005, DR3305, and PZ‐51, is a synthetic organoselenium compound first synthesized in 1924. Its biological significance was recognized decades later when it was demonstrated to mimic the activity of selenium‐dependent antioxidant enzyme glutathione peroxidase (GPx) [1]. GPx is a component of the endogenous antioxidant defense in biological systems. It is also a selenoenzyme in which selenium, present as selenocysteine at the catalytic site, is essential for the reduction of hydroperoxides using glutathione (GSH) as the electron donor [2]. GPx protects cells against oxidative damage by catalyzing the reduction of hydrogen peroxide and phospholipid hydroperoxides, thereby preserving membrane integrity and cellular homeostasis. The identification of phospholipid hydroperoxide GPx (GPx4), which protects biological membranes, highlighted the critical role of selenium‐dependent enzymes in preventing lipid peroxidation [3].

In 1984, Ebselen was demonstrated to catalyze the reduction of hydroperoxides through a GPx‐like mechanism and defined as having a thiol‐dependent catalytic reduction activity, establishing its GPx‐like antioxidant properties [4, 5]. Since then, several investigations preclinically and clinically started to evaluate its therapeutic potential in disorders mediated primarily by oxidative stress. Ebselen has been investigated for nearly a century, evolving from a synthetic organoselenium compound to a promising multitarget therapeutic candidate [6].

There are multiple complementary mechanisms through which Ebselen modulates oxidative stress, including the maintenance of redox homeostasis and inhibition of pro‐oxidant signaling pathways. It interacts with the thioredoxin (Trx) system and cellular thiols, reduces peroxynitrite, scavenges reactive oxygen and nitrogen free radicals, and reversibly modifies reactive cysteinyl residues in target proteins, thereby regulating their activity. For example, in silico studies have shown that Ebselen interacts with the critical cysteinyl residue (C218) of inositol monophosphatase (IMPase), suggesting that enzyme inhibition occurs through oxidation of this catalytic residue [7]. Ebselen was also reported to induce the activation of Nrf2 through oxidizing the cysteinyl residues in Keap1, allowing Nrf2 to transcriptionally activate the expression of antioxidant enzymes [8]. Consequently, Ebselen’s thiol‐modifying activity is now considered a major determinant of its antioxidant, anti‐inflammatory, antimicrobial, and cytoprotective effects [9].

Structurally, Ebselen contains a selenium atom incorporated within a benzisoselenazolone heterocycle, which confers its unique redox reactivity. Its antioxidant activity is mediated through a thiol‐dependent catalytic cycle. Upon the reaction with GSH, cysteine, or protein thiol groups, Ebselen forms a reversible selenyl–sulfide (Se–S) intermediate [10]. This intermediate enables the catalytic reduction of hydrogen peroxide and lipid hydroperoxides to water and alcohol, mimicking the activity of GPx activity and thus limiting lipid peroxidation and free radical production [11, 12].

Ebselen has attracted considerable interest and emerged as a promising drug candidate for the treatment of diverse pathological conditions. Interestingly, Ebselen has been evaluated clinically in various nonhepatic conditions, including Meniere’s disease, bipolar disorder, and sensorineural hearing loss [13–17]. These trials demonstrated the tolerability and safety of Ebselen, supporting the applicability of its use in humans and strengthening its repurposing potential for liver diseases. More recently, Ebselen has attracted considerable attention as a drug repurposing candidate for the treatment of infections caused by multidrug‐resistant microorganisms owing to its ability to inhibit essential thiol‐dependent enzymes involved in microbial survival [18]. It has also been repurposed for the therapeutic targeting of COVID‐19 and other respiratory viral infections owing to its antiviral activity and its ability to modulate host oxidative stress responses [19]. Clinical investigations have also explored the potential of Ebselen in neuropsychiatric disorders. In patients with treatment‐resistant depression, adjunctive Ebselen altered emotional processing and brain neurochemistry, supporting its biological activity in psychiatric disorders and warranting further evaluation of its therapeutic potential [20, 21]. Ebselen has also been proposed as an antidote for toxic‐metal exposure. For example, in mercury intoxication, Ebselen was shown to lower the body burden of Hg, where it mitigates mercury‐induced oxidative stress and preserves the function of selenoenzymes. Ebselen is metabolized to an intermediate with a ─SeH (selenol) functional group, which has a greater affinity to electrophilic Hg than the available therapeutic agents. The observed protective effects of Ebselen against MeHg^+^ indicate its potential as a therapeutic agent to treat MeHg^+^ overexposure [22]. Furthermore, Ebselen has demonstrated cardioprotective effects in experimental models of iron‐overloaded thalassemia by attenuating iron‐induced oxidative stress, preserving mitochondrial function, and improving cardiomyocyte survival, further supporting its therapeutic potential in diseases characterized by redox imbalance [23]. In addition to Ebselen applications in human medicine, its therapeutic potential has recently been extended to veterinary medicine. It has been evaluated as a repurposed anthelmintic agent against gastrointestinal nematode infection in small ruminants, highlighting its potential to address drug resistance in veterinary parasitology [24]. These diverse applications underscore the versatility of Ebselen as a multitarget therapeutic compound across both human and veterinary medicine.

**Figure 1:**
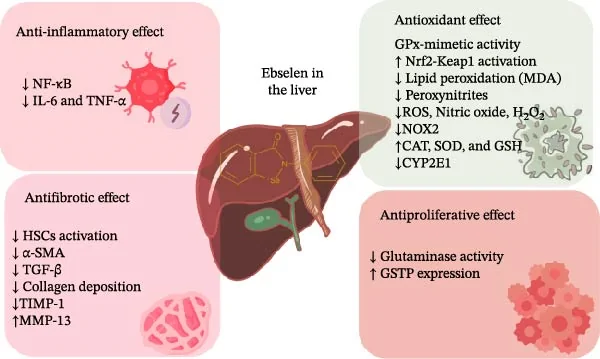
Molecular mechanisms through which Ebselen exerts it hepatoprotectivity.

The liver is highly susceptible to damage caused by oxidative stress due to its crucial functions in xenobiotic metabolism, lipid homeostasis, and immune regulation. Excessive oxidative damage and the resulting aggravated inflammatory response are central mechanisms involved in several liver diseases, including drug‐induced liver injury, alcoholic liver disease, nonalcoholic fatty liver disease (NAFLD), and ischemic reperfusion injury [25, 26]. Given that selenium hepatoprotective effects were demonstrated to counteract necrotic liver degeneration, both natural and synthetic organoselenium compounds have been evaluated for their potential protective effects [27–31]. Ebselen is recognized for its antioxidant and anti‐inflammatory properties and has attracted significant attention as a hepatoprotective agent in both in vitro and in vivo models by inhibiting lipid peroxidation and preserving mitochondrial function. Ebselen has been demonstrated to influence signaling pathways related to apoptosis and fibrosis. The multitarget pharmacological profile has established Ebselen as a promising therapeutic agent for various oxidative stress‐ and inflammation‐induced disorders [32]. Figure 1 represents the proposed mechanisms of action of Ebselen in the liver. This multifaceted role identifies Ebselen as a strong candidate for both preventive and therapeutic effects on hepatocellular injury. This review outlines the existing evidence regarding the role of Ebselen in liver pathologies by summarizing experimental data and emphasizing mechanisms of action.

### 1.1. Safety and Toxicological Profile of Ebselen

Ebselen use is generally associated with a favorable safety profile in both preclinical and clinical studies. Compared with many other organoselenium compounds, ebselen exhibits relatively low toxicity at therapeutic doses, where its toxic effect depends on the dose, duration of exposure, route of administration, and species. The supratherapeutic doses may induce toxicity through excessive interaction with the oxidation of cellular thiols, resulting in thiol depletion, oxidative imbalance, lipid peroxidation, and inhibition of sulfhydryl‐dependent enzymes. Such toxic effects have been associated with the inhibition of enzymes including δ‐aminolevulinate dehydratase (δ‐ALA‐D), Na^+^/K^+^‐ATPase, and lactate dehydrogenase [33]. In an acute toxicity study, Meotti et al. evaluated the potential renal and hepatic toxicity of several organochalcogens, including Ebselen, in rats and mice. Ebselen exhibited moderate, route‐dependent toxicity, with LD_50_ values of ~400 µmol/kg in rats and 340 µmol/kg in mice following intraperitoneal administration, whereas subcutaneous administration resulted in lower toxicity (>500 µmol/kg). Although detailed biochemical and histopathological analyses were not extensively described, the authors indicated possible hepatic and renal effects at higher doses, suggesting that excessive exposure to Ebselen could induce organ toxicity, and systemic toxicity is strongly influenced by the route of administration [34].

Another study also evaluated the cytotoxic and genotoxic effects of Ebselen in isolated human leukocytes as a preliminary model for toxicity screening. Ebselen exhibited concentration‐dependent cytotoxicity, significantly reducing leukocyte viability only at 50 μM, while lower concentrations (5–10 μM) had no significant effect. Similarly, DNA damage was detected only at the highest concentration tested, indicating limited genotoxicity under the experimental conditions. The paper also suggested that Ebselen‐induced toxicity may result from the oxidation of critical thiol‐containing proteins and impairment of mitochondrial function. The method was proposed as an early screening tool for evaluating the safety of organochalcogen compounds [35]. Ebselen does not accumulate extensively in tissues under normal conditions, a characteristic that may contribute to its relatively low systemic toxicity compared with that of other organoselenium compounds. Following absorption, Ebselen undergoes rapid thiol‐dependent metabolism through reversible interactions with GSH and protein thiols, forming selenenyl sulfide intermediates and selenol‐containing metabolites. Because its metabolism relies on reactions with cellular thiols, excessive doses may disrupt thiol homeostasis and contribute to adverse effects [36].

An important consideration is the concentration‐dependent effect of Ebselen on mitochondrial function. In isolated rat liver mitochondria, low concentrations promoted calcium‐dependent opening of the mitochondrial permeability transition pore, an effect mediated by oxidation of protein thiol groups and linked to ebselen’s GPx‐mimetic activity. At higher concentrations, ebselen inhibited pore opening and thiol oxidation [37]. This duality suggests that the Ebselen biological effect is dependent on the concentration achieved locally in the liver tissue, a factor that should be considered when evaluating dosing and hepatotoxicity. Ebselen administration was also shown to deplete hepatic nonprotein thiols (NPSHs) and increase lipid peroxidation in rat liver, independent of any coexposure, supporting thiol depletion as a mechanism of Ebselen‐induced hepatotoxicity [38]. Ebselen exhibits a favorable safety profile characterized by low toxicity at therapeutically relevant doses. Although excessive exposure may lead to thiol‐dependent adverse effects, its wide therapeutic window supports its continued investigation as a therapeutic agent.

### 1.2. Ebselen in Liver Fibrosis

Persistent liver injury can lead to the development of liver fibrosis, a pathological process characterized by excessive collagen deposition and extracellular matrix components, resulting in the replacement of healthy liver parenchyma with fibrotic scar tissue. This process is primarily driven by the activation of hepatic stellate cells (HSCs), the principal fibrogenic cells [39, 40]. Oxidative stress is a key contributor to the development of liver fibrosis. Oxidative injury to hepatocytes promotes mitochondrial generation of reactive oxygen species (ROS), which stimulate lipid peroxidation and activate inflammatory and fibrogenic pathways [41]. Owing to its antioxidant and redox‐modulating effects, Ebselen has been investigated as a potential antifibrotic agent. In a model of carbon tetrachloride (CCL4)‐induced liver fibrosis, Ebselen treatment significantly improved liver histopathology and reduced collagen deposition. The histological improvement was accompanied by a reduced level of transcribed transforming growth factor‐β (TGF‐β) and a significant reduction in procollagen I and procollagen III mRNA levels by 35% and 40%, respectively, compared with the untreated fibrotic control group. Collagen turnover is tightly regulated by matrix metalloproteinases (MMPs) and their endogenous inhibitors, tissue inhibitors of metalloproteinases (TIMPs). In the CCL4‐induced liver fibrosis, Ebselen treatment modulated the expression of these regulators, thereby promoting extracellular matrix remodeling. Specifically, Ebselen downregulated the mRNA expression of TIMP‐1 while increasing the expression of MMP‐13 compared to the untreated CCL4 group, suggesting enhanced degradation of accumulated collagen. Cytochrome P450 2E1 (CYP2E1), a major source of ROS during xenobiotic metabolism, is known to contribute to oxidative stress and the progression of hepatic fibrosis [42]. Ebselen treatment significantly reduced CYP2E1 gene expression by ~25% compared with the fibrotic group, further supporting the antioxidant effect of Ebselen [43]. Molecular targets through which Ebselen exerts its antifibrotic effects are summarized in Table 1.

**Table 1:** A summary of Ebselen protective mechanisms in liver fibrosis.

Dosage	Hepatotoxic agent	Species	Protective mechanisms	References
0.4 mg/mL, p.o for 3 weeks	Carbon tetrachloride	Fischer rats	↓Collagen ↓TGF‐β1 ↓Procollagen I and III ↓TIMP‐1 ↑MMP‐13 ↓CYP2E1 ↓G3PDH	[43]
5 mg/kg, p.o for 28 days.	Arsenic	Rats	↓Bilirubin, ALT, and ALP ↓MDA and NO ↑GSH, SOD, CAT, GR, and GPx ↓IL‐6, TNF‐α, and MPO ↓TGF‐β1 and EGF	[44]
10 mg/kg, p.o for 16 weeks.	High‐fat diet	C57BL/6 mice	↓TG, TC, and LDL‐C ↑HDL‐C ↑PPARα, CPT1α, ACOX1, UCP2, and PGC1α ↓TNF‐α, IL‐1β, and IL‐6 ↑PI3K/Akt ↓TLR4/JNK	[45]

In another experimental study, Ebselen showed broad hepatoprotective effects by preserving both hepatic function and histological architecture in arsenic‐intoxicated rats. Histopathological examination revealed that Ebselen attenuated hepatic injury, as evidenced by reduced lipid accumulation, inflammatory cell infiltration, and hepatocyte necrosis. These protective effects were accompanied by a significant improvement in the hepatic redox status. Specifically, Ebselen restored GSH levels while significantly reducing malondialdehyde (MDA) and nitric oxide (NO) concentrations. Moreover, Ebselen enhanced the activities of several endogenous antioxidant enzymes, including superoxide dismutase (SOD), catalase (CAT), glutathione reductase (GR), and GPx, further demonstrating its potent antioxidant capacity.

Apoptosis has been recognized as an important contributor to the progression of liver fibrosis. Apoptotic hepatocytes release apoptotic bodies that activate hepatic macrophages, which subsequently promote HSC activation [46]. In the same study, Ebselen significantly reduced the expression of the proapoptotic markers caspase 3 and Bax, indicating a marked antiapoptotic effect. Furthermore, Ebselen decreased the expression of the profibrotic mediators both TGF‐β1 and epidermal growth factor (EGF) compared with the untreated disease group, suggesting that its hepatoprotective effects extend beyond oxidative stress attenuation to include modulation of apoptosis and fibrogenic signaling pathways [44]. Ebselen has also demonstrated therapeutic potential in NAFLD by improving hepatic lipid metabolism and reducing steatosis. Mechanistically, this effect was associated with the activation of the PI3K/Akt signaling pathway, which promotes lipid homeostasis and cell survival, together with the inhibition of the TLR4/JNK inflammatory pathway, thereby attenuating hepatic inflammation and lipid accumulation [45].

Despite the numerous preclinical studies demonstrating promising pharmacological effects for Ebselen, much of this evidence is derived from in vitro studies and animal models. These findings provide important mechanistic insights but may not be directly applicable for clinical applications because of the variations in pharmacokinetics and disease pathophysiology. The use of different experimental models representing distinct mechanisms of liver injury complicates direct comparisons between studies. As a result, it remains unclear whether Ebselen exerts consistent antifibrotic effects across diverse etiologies of chronic liver disease.

### 1.3. Ebselen in Hepatocellular Carcinoma (HCC)

Hepatocellular carcinogenesis is a multistep process through which normal hepatocytes undergo malignant transformation, leading to HCC, the most common primary liver malignancy. HCC typically develops due to chronic liver injury and persistent inflammation, particularly in patients with chronic viral hepatitis, cirrhosis, and other chronic liver diseases [47]. Altered metabolic reprogramming has been closely linked to tumor initiation and progression. For instance, enhanced glutamine metabolism (glutaminolysis) provides proliferating tumor cells with energy and biosynthetic precursors and is associated with increased activities of glutaminase and glutamine dehydrogenase [48]. Consequently, glutaminase inhibition has emerged as a promising therapeutic strategy for suppressing tumor cellular proliferation in various preclinical cancer models [49]. In a kinetic characterization‐based study, Ebselen was identified as a potent glutaminase inhibitor. Ebselen inhibited glutaminase in a time‐ and concentration‐dependent pseudo‐first‐order mechanism and exhibited a 10‐ to 1500‐fold greater affinity than the established glutaminase inhibitors 6‐diazo‐5‐oxo‐L‐norleucine (DON) and bis‐2‐(5‐phenylacetamido‐1,2,4‐thiadiazol‐2‐yl)ethyl sulfide (BPTES). Ebselen also showed a more than 100‐fold higher inhibitory efficiency [50]. Table 2 summarizes the signaling pathways targeted by Ebselen in HCC.

**Table 2:** Ebselen protective molecular mechanisms in HCC.

Dosage	Species	Protective mechanisms	References
1 mg/mL	HepG2 cells	↓DPPH ↓Cell proliferation	[51]
50, 75, and 100 µM for 24 h	HepG2 cells	↓Cell viability ↑Apoptotic cells ↓Thiols	[52]
810 nmol, transdermal	Mice	↓TBARS ↓H_2_O_2_ ↑GST and NQO1 ↑GSTP1	[53]
0.71–365 µM	Hepa 1c1c7	↑QR and GST ↓Cell proliferation	[54]
5 mg/kg, orally, 28 weeks	Male Fisher 344 rats	↓AFP ↓8‐OHdG	[55]

In addition to altered glutamine metabolism, the dysregulated Trx system is implicated in the induction of malignant tumors. Trx reductase (TrxR), a selenium‐dependent oxidoreductase, is a regulator of cellular redox homeostasis and promotes tumor cell survival, proliferation, DNA synthesis, and resistance to apoptosis. TrxR is frequently overexpressed in a variety of malignancies, including HCC, where its elevated activity contributes to tumor progression and resistance to anticancer therapies [56]. Therefore, TrxR has emerged as a promising pharmacological target for cancer treatment [57]. Among its components, TrxR1 is frequently overexpressed in numerous human malignancies, including HCC, where elevated expression has been associated with enhanced tumor cell proliferation and resistance to apoptosis. Consequently, TXNRD1 has emerged as an attractive therapeutic target for anticancer drug development [58]. The anticancer properties of Ebselen [59, 60] have stimulated the development of novel Ebselen‐derived hybrid compounds [61–63]. One such compound combines the pharmacophore of Ebselen with a stilbene scaffold exhibited potent antiproliferative effects against multiple human cancer cell lines by inducing cell cycle arrest and effectively inhibiting TrxR activity [64]. Similarly, the benzoselenazole–stilbene hybrid incorporating the pharmacophore of both resveratrol and Ebselen exhibited an inhibition of cancer cell growth through suppression of TrxR activity, increased oxidative stress, and induction of apoptosis [65]. Furthermore, other Ebselen derivatives, including Ebselen selenocyanate and Ebselen diselenide, exhibited GPx‐like antioxidant activity together with cytotoxic effects against HepG2 HCC cells, suggesting that structural modification of Ebselen may enhance its antitumor potential [51].

The antitumor effect of Ebselen has also been investigated in human HCC cell lines. In an in vitro study, Ebselen significantly reduced cell viability in a concentration‐ and time‐dependent manner. The reduction in cell viability was accompanied by the induction of apoptotic death in HepG2 cells, as demonstrated by the increased number of TUNEL‐positive cells and characteristic apoptotic morphological alterations, including cell shrinkage, membrane blebbing, chromatin condensation, and DNA fragmentation. These effects were further associated with the depletion of intracellular GSH concentration [52]. Ebselen has also demonstrated chemopreventive properties through the induction of phase II enzymes. These enzymes, including glutathione S‐transferases (GSTs) and UDP‐glucuronosyl transferases (UGTs), play an essential role in the detoxification of carcinogens and the maintenance of cellular antioxidant defense, thereby reducing the susceptibility to carcinogenesis [66, 67]. Consistent with the well‐established chemopreventive properties of selenium and selenium‐rich compounds [68, 69], Ebselen was shown to increase the activity of quinone reductase (QR) and GST in liver epithelial cells, exhibiting an enzyme‐inducing potency comparable to that of ter‐butylhydroquinone, a well‐known inducer of phase II detoxification enzymes [53]. Similar induction of QR and GST activity, accompanied by inhibition of cell growth, was observed in Hepa 1c1c7 cells [54]. Ebselen was also shown to have protective effects against aflatoxin B1 (AFB1)‐induced hepatocarcinogenesis. Alpha‐fetoprotein (AFP) is synthesized by the liver, and high expression is correlated with the onset of HCC, and therefore, it is a marker of hepatic tumors [70]. AFP was significantly downregulated following Ebselen treatment in AFB1‐exposed animals. Furthermore, Ebselen reduced the formation of the mutagenic AFB_1_–DNA adduct (AFB_1_–FAPY) when compared to the AFB_1_ group. It also attenuated AFB_1_‐induced oxidative DNA damage, as evidenced by the decreased level of 8‐hydroxy‐2′‐deoxyguanosine (8‐OHdG), the oxidized form of DNA [55].

### 1.4. Ebselen in Liver Injury

Liver injury results from acute or chronic insults that compromise the functional and structural integrity of hepatocytes. These insults arise from a variety of etiologies, including drug‐induced toxicity, viral infections, alcohol abuse, metabolic disorders, ischemia‐reperfusion injury, and exposure to environmental toxins. Regardless of the underlying cause, liver injury is characterized by oxidative stress, inflammation, and hepatocellular damage that overwhelm the liver’s remarkable capacity for repair and regeneration. If the injurious stimulus persists, progressive pathological changes, including hepatitis, steatosis, hepatocytes necrosis, fibrosis, and ultimately cirrhosis, may develop, increasing the risk of liver failure [71]. Selenium is a dietary trace element that plays a role in maintaining cellular redox homeostasis through its incorporation into selenoproteins, including GPxs and Trx reductases. These enzymes protect cells against oxidative damage by reducing lipid peroxidation, limiting protein oxidation, and DNA damage, thereby preserving cellular integrity. Selenium is not synthetized endogenously, and therefore, adequate dietary intake is required to maintain optimal selenoprotein function [72]. Ebselen, as one of the selenium compounds, has shown hepatoprotective effects against ethanol‐induced liver injury. In an in vitro model, Ebselen protected hepatocytes from ethanol‐induced cytotoxicity, improved cell viability, and restored GSH level and SOD activity. Apoptotic cell death in hepatocytes was remarkably lower in cells incubated in Ebselen, an effect accompanied by modulation of ferroptosis [73]. The hepatoprotective effects of Ebselen were further confirmed in vivo. In a rat model of ethanol‐induced hepatic injury. Ebselen significantly attenuated pathological alterations, decreased inflammatory necrotic foci, and neutrophils infiltrations [74]. In addition to its antioxidant and anti‐inflammatory effects, Ebselen also demonstrated a vasoprotective effect by exerting a vasodilative effect against alcohol‐induced constriction of the hepatic vasculature, resulting in a 50% to 75% reduction in portal pressure after ethanol infusion [75]. In addition, Ebselen restored ethanol‐induced reduction in GPx and δ‐ALA‐D activities, as well as NPSH levels [76].

The hepatoprotective effect of Ebselen was demonstrated in several experimental models of acute liver injury. In a *Propionibacterium acnes* and lipopolysaccharide (LPS)‐induced hepatitis model, Ebselen suppressed macrophage‐induced inflammatory injury in the liver. The administration of Ebselen attenuated macrophage‐mediated hepatic injury by inhibiting hepatocellular necrotic degeneration and suppressing the production of proinflammatory cytokines released from activated macrophages, thereby reducing macrophage activation [77]. Similarly, immunomodulatory effects of Ebselen were also evaluated in a model of immune‐mediated hepatitis induced by concanavalin A (ConA) and galactosamine (GalN) injected separately. Pretreatment with Ebselen significantly inhibited the release of inflammatory cytokines and the induction of apoptosis [78]. Ebselen also protected Kupffer cells against free radical‐induced reoxygenation injury by inhibiting NADPH oxidase activity, thereby decreasing the production of superoxide anions and NO [79]. Consistent with these findings, it was demonstrated that Ebselen protected against D‐galactosamine/endotoxin‐induced hepatitis when administered 1 h before the toxin exposure [80]. In a related model of D‐galactosamine/endotoxin‐induced shock, Ebselen reduced transaminases activities and attenuated cellular damage [81].

Ebselen has also shown protective effects in drug‐induced hepatotoxicity. Owing to its GSH‐peroxidase‐mimetic activity, Ebselen ameliorated Paracetamol‐induced liver injury by limiting the formation of the reactive metabolite, NAPQI [82]. In another study, Ebselen preserved the intracellular GSH level and reduced lipid peroxidation following paracetamol exposure [83]. Moreover, Ebselen restored the activity of delta‐aminolevulinic acid dehydratase (δ‐ALA‐D), a thiol‐dependent enzyme essential for heme biosynthesis, whose activity is highly susceptible to oxidative modification due to its redox‐sensitive cysteine residues [84].

Among the various mediators implicated in hepatocellular injury, hydrophobic bile acids, particularly deoxycholic acid (DCA), represent a major source of cholestatic liver damage. DCA exhibits potent detergent‐like properties that disrupt cellular membrane integrity [85]. As a hydrophobic bile acid, it exerts cytotoxic effects by solubilizing membrane lipids, leading to increased membrane permeability, mitochondrial dysfunction, and ROS generation and promoting oxidative stress‐mediated hepatocellular injury [86]. Ebselen treatment has been shown to protect against DCA‐induced liver injury in rats by preserving hepatic drug‐metabolizing capacity and improving liver function. Ebselen treatment also inhibited DCA‐induced elevation of serum ALP and ALT, maintaining their levels close to 1.4‐ and 1.9‐fold those of the control values, respectively. In addition, Ebselen prevented DCA‐induced reduction of hepatic cytochrome P450 enzymes CYP1A1 and CYP3A2, preserving their protein expression levels at 86% and 65% of the control, respectively [87]. Table 3 compiles experimental data of Ebselen across various liver injury models.

**Table 3:** Principal antioxidant and protective mechanisms of Ebselen in liver injury models.

Dosage	Species	Protective mechanisms	References
2.0 μM	NCTC clone 1469	↑GPX4 and SLC7A11 ↓ACSL4	[73]
50 mg/kg, orally for 4 weeks	Wistar rats	↓ALT ↓4‐hydroxynonenal ↓Nitrate/nitrite ↓NF‐κB activity	[74]
10 or 30 µM	Rats	↓Portal pressure ↓LDH	[75]
5 mg/kg, s.c for 30 days	Mice	↓GPX4 ↓δ‐ALA‐D	[76]
300 mg/kg, p.o for 2 days	Rats	↓TNF‐α and IL‐18 ↓IFNγ‐inducing factor ↓IFNγ ↓ROS ↓ALT ↓Necrosis	[77]
600 mg/kg	Rats	↓Sorbitol dehydrogenase and SGOT SGPT	[80]
50, 100 µM	Hepatocytes	↓LDH ↓LPO ↑GSH	[83]
100 µmol/kg, s.c	Wistar rats	↓δ‐ALA‐D	[84]
30 mg/kg, orally, for 10 days	Rats	↓ALT and ALP ↑CYP1A1 and CYP3A2	[87]
50–200 mg/kg, 4 times/day for 3 days	Sprague‐Dawley rats	↓Bilirubin	[88]
50 mg/kg, orally	Rats	↓ALT ↓ROS	[89]
0.1–100 µM for 3, 12, 24 h.	Rat Kupffer cells	↓NO, TNF‐α, iNOS, and COX‐2 ↓NF‐kB ↓p‐JNK	[90]
25 µmol/L	Hepatocytes	↓ROS ↓Apoptosis	[91]
50 µmol/kg, 5 min before I/R	Rats	↓ALT and AST ↓MDA ↑GSH ↓Bax and cytochrome C ↓Bcl‐2	[92]

A proof‐of‐concept study also evaluated the protective effect of combined nutritional and pharmacological intervention in pancreatitis‐induced organ dysfunction. The nutritional supplementation with the antioxidant, Ebselen, together with the dietary fiber, ethylhydroxyethyl cellulose (EHEC), reduced bilirubin level compared with the EHEC alone group, attenuating pancreatitis‐associated hepatic dysfunction [88]. Although the liver outcome is relatively simple, since the study is focused on multi‐organ dysfunction, the liver findings are supportive rather than central, supporting the beneficial effects of Ebselen on liver function during systemic inflammatory conditions. Similar protective antioxidant effects of Ebselen were further highlighted in LPS‐induced liver injury, where Ebselen restored the hepatic antioxidant balance and decreased ROS production [89]. Additionally, Ebselen significantly suppressed LPS‐induced production of inflammatory mediators in Kupffer cells primarily by inhibiting JNK phosphorylation and preventing NF‐ κB nuclear translocation, which supports a mechanistic basis for Ebselen’s anti‐inflammatory action in hepatic macrophages and its potential in protecting against endotoxin‐mediated liver injury [90]. Ebselen also attenuated lipid peroxidation in Adriamycin‐induced hepatic toxicity [93]. The potent antioxidant activity of Ebselen has also been shown in an experimental model of ischemia‐reperfusion (I/R) injury. Ebselen treatment conferred hepatoprotection by preserving intracellular GSH, suppressing the generation of ROS in hepatocytes, and attenuating apoptosis by inhibiting mitochondrial permeability transition [91]. Ebselen also showed potential protective effects against I/R injury by mitigating oxidative stress, lipid peroxidation, and associated inflammatory responses [94].

A newer structural analog, 2‐selenium‐bridged β‐cyclodextrin, exhibits comparable hepatoprotective efficacy, preserving liver integrity by modulating redox‐sensitive inflammatory pathways. This activity reflects the redox‐active nature of the selenium bridge, similar to that in Ebselen, which underpins both compounds’ antioxidant and anti‐inflammatory actions. These parallels highlight the selenium moiety as the critical pharmacophore responsible for their protective effects. Several technologies are commonly used to improve the delivery of poorly water‐soluble drugs and enhance their solubility, dissolution, oral absorption, and tissue exposure. These include lipid‐based formulations, self‐emulsifying drug delivery systems (SEDDS), polymeric nanoparticles, solid dispersions, and cyclodextrin inclusion complexes. The structural modification of Ebselen by incorporating a β‐cyclodextrin framework appears to enhance therapeutic potential by improving water solubility and overcoming the limited bioavailability often associated with Ebselen [92]. Other formulation‐based studies further support the need to consider drug delivery in the drug development of Ebselen. This includes biopolymeric nanocapsules for topical antifungal therapy [95], orally administered nanoparticles for ulcerative colitis [96], and polymer‐engineered liposomes for cancer treatment [97]. These studies consistently demonstrate that formulation strategies can improve Ebselen’s solubility, stability, tissue targeting, and therapeutic efficacy. However, whether similar Ebselen delivery platforms can improve hepatic targeting and intrahepatic bioavailability should be addressed in future studies.

## 2. Conclusion

Ebselen is a promising multifunctional synthetic selenoorganic compound with therapeutic potential due to its potent antioxidant effect. It has been recognized as a GPx mimetic. It exerts its GPx‐mimic activity through a nucleophilic attack on the thiol group of the amino acid by its electrophilic selenium center. Its redox‐modulating property is attributed to its capability of interacting with the Trx pathway and various thiol‐containing proteins. Through scavenging free radicals and suppressing lipid peroxidation and oxidative inflammation, Ebselen exerts cellular protective effects. In liver disorders, where oxidative stress dominates the causative etiology, Ebselen emerges as a hepatoprotictive agent. Experimental studies constantly showed attenuated oxidative damage and modulated redox‐sensitive enzymes.

Despite extensive preclinical evidence demonstrating the hepatoprotective and antifibrotic effects of Ebselen in preclinical experimental models of liver injury, no clinical trials have yet evaluated its efficacy or safety in patients with liver diseases, highlighting a significant translational gap, an unestablished clinical relevance, and the need for well‐designed Phase I and II clinical studies. A key translational consideration is pharmacokinetics. Ebselen is orally bioavailable and has been evaluated in humans for other indications; however, its absorption, distribution, metabolism, and elimination may be altered in patients with chronic liver disease, particularly in advanced fibrosis or cirrhosis. Since hepatic dysfunction can influence drug metabolism and systemic exposure, dedicated pharmacokinetic studies in liver disease populations are required before therapeutic dosing can be reliably defined.

The doses used in experimental liver fibrosis models cannot be directly extrapolated to humans because of interspecies differences in metabolism, bioavailability, and disease biology. Moreover, the optimal therapeutic window for antifibrotic efficacy is unknown, including whether Ebselen would be more effective during early inflammatory stages or established fibrosis. Therefore, dose–response studies and pharmacodynamic biomarkers are needed to determine how the preclinical benefits of Ebselen can be translated into clinical outcomes.

Despite growing interest in Ebselen as a therapeutic agent, formulation research remains limited. To date, only a few studies have explored advanced delivery systems, including biopolymeric nanocapsules for topical antifungal therapy, orally administered nanoparticles for ulcerative colitis, and polymer‐engineered liposomes for cancer treatment. These studies consistently demonstrate that formulation strategies can improve Ebselen’s solubility, stability, tissue targeting, and therapeutic efficacy. However, no comparable delivery systems have yet been developed or evaluated for liver diseases, representing a significant gap in the translational development of Ebselen. Future studies should investigate liver‐targeted nanocarriers, such as lipid nanoparticles, polymeric nanoparticles, or ligand‐modified delivery systems, to enhance hepatic accumulation and optimize antifibrotic efficacy.

Safety is another important consideration. Ebselen has generally been reported to be well tolerated in previous clinical investigations, but its long‐term safety in patients with chronic liver injury remains unclear. Liver fibrosis patients may require prolonged treatment and may have comorbidities and concurrent medications that increase the risk of adverse effects or drug–drug interactions. Therefore, future studies should carefully assess liver enzyme changes, systemic toxicity, and safety during repeated or long‐term administration of Ebselen.

In addition, although a large number of studies have investigated the therapeutic potential of Ebselen, a considerable proportion of the studies was conducted several years ago. While these studies form the basis of the fundamental research elucidating mechanisms of Ebselen, advanced research on signaling pathways and molecular mechanisms by using advanced contemporary techniques is warranted. This may delineate the context‐dependent interacting mechanisms of Ebselen with redox systems in order to expand its therapeutic relevance and minimize undesired effects.

Moreover, the most obvious limitation is the lack of studies. The available studies consistently suggest that Ebselen attenuates liver damage, but the evidence base remains limited, with few published studies investigating this indication. Consequently, the current findings should be interpreted as preliminary rather than conclusive. The preclinical studies provide mechanistic insights, but their predictive value for clinical efficacy remains uncertain because experimental liver disease models do not fully recapitulate the complexity and heterogeneity of human liver diseases. Furthermore, disease heterogeneity may limit the generalizability of the findings obtained from controlled experimental models.

Consequently, future research should focus on addressing the current limitations to strengthen the evidence base and improve the generalizability of the findings.

## Funding

This research was supported by the United Arab Emirates University (Grant 12R121).

## Conflicts of Interest

The authors declare no conflicts of interest.

## Data Availability

Data sharing is not applicable to this article as no datasets were generated or analyzed during the current study.

## References

[1] Parnham M. J. and Sies H. , The Early Research and Development of Ebselen, Biochemical Pharmacology. (2013) 86, no. 9, 1248–1253, 10.1016/j.bcp.2013.08.028. 24012716

[2] Flohe L. , Günzler W. A. , and Schock H. H. , Glutathione Peroxidase: A Selenoenzyme, FEBS Letters. (1973) 32, no. 1, 132–134, 10.1016/0014-5793(73)80755-0. 4736708

[3] Ursini F. , Maiorino M. , Valente M. , Ferri L. , and Gregolin C. , Purification From Pig Liver of a Protein Which Protects Liposomes and Biomembranes From Peroxidative Degradation and Exhibits Glutathione Peroxidase Activity on Phosphatidylcholine Hydroperoxides, Biochimica et Biophysica Acta. (1982) 710, no. 2, 197–211, 10.1016/0005-2760(82)90150-3. 7066358

[4] Müller A. , Cadenas E. , Graf P. , and Sies H. , A Novel Biologically Active Seleno-Organic Compound—1: Glutathione Peroxidase-Like Activity In Vitro and Antioxidant Capacity of PZ. 51 (Ebselen), Biochemical Pharmacology. (1984) 33, no. 20, 3235–3239, 10.1016/0006-2952(84)90083-2. 6487370

[5] Wendel A. , Fausel M. , Safayhi H. , Tiegs G. , and Otter R. , A Novel Biologically Active Seleno-Organic Compound—II. Activity of PZ 51 in Relation to Glutathione Peroxidase, Biochemical Pharmacology. (1984) 33, no. 20, 3241–3245, 10.1016/0006-2952(84)90084-4. 6487371

[6] Nogara P. A. , Pereira M. E. , Oliveira C. S. , Orian L. , and da Rocha J. B. T. , Lippolis V. , Santi C. , Lenardão E. J. , and Braga A. L. , The Long Story of Ebselen: From About One Century of Its Synthesis to Clinical Trials, Chalcogen Chemistry: Fundamentals and Applications, 2023, The Royal Society of Chemistry.

[7] Barbosa N. V. , Nogueira C. W. , Nogara P. A. , de Bem A. F. , Aschner M. , and Rocha J. B. T. , Organoselenium Compounds as Mimics of Selenoproteins and Thiol Modifier Agents, Metallomics. (2017) 9, no. 12, 1703–1734, 10.1039/C7MT00083A. 29168872

[8] Orian L. and Toppo S. , Organochalcogen Peroxidase Mimetics as Potential Drugs: A Long Story of a Promise Still Unfulfilled, Free Radical Biology and Medicine. (2014) 66, 65–74, 10.1016/j.freeradbiomed.2013.03.006. 23499840

[9] Kumar M. , Garg S. , and Berlicki Ł. , Molecular Basis of Ebselen Biological Activity, Future Medicinal Chemistry. (2026) 18, no. 11, 1465–1480, 10.1080/17568919.2026.2658832. 41988750 PMC13228979

[10] Sands K. N. and Back T. G. , Key Steps and Intermediates in the Catalytic Mechanism for the Reduction of Peroxides by the Antioxidant Ebselen, Tetrahedron. (2018) 74, no. 38, 4959–4967, 10.1016/j.tet.2018.05.027.

[11] Azad G. K. and Tomar R. S. , Ebselen, a Promising Antioxidant Drug: Mechanisms of Action and Targets of Biological Pathways, Molecular Biology Reports. (2014) 41, no. 8, 4865–4879, 10.1007/s11033-014-3417-x. 24867080

[12] Noguchi N. , Ebselen, a Useful Tool for Understanding Cellular Redox Biology and a Promising Drug Candidate for Use in Human Diseases, Archives of Biochemistry and Biophysics. (2016) 595, 109–112, 10.1016/j.abb.2015.10.024. 27095225

[13] Kil J. , Pierce C. , Tran H. , Gu R. , and Lynch E. D. , Ebselen Treatment Reduces Noise Induced Hearing Loss via the Mimicry and Induction of Glutathione Peroxidase, Hearing Research. (2007) 226, no. 1-2, 44–51, 10.1016/j.heares.2006.08.006. 17030476

[14] Kil J. , Lobarinas E. , and Spankovich C. , et al.Safety and Efficacy of Ebselen for the Prevention of Noise-Induced Hearing Loss: A Randomised, Double-Blind, Placebo-Controlled, Phase 2 Trial, The Lancet. (2017) 390, no. 10098, 969–979, 10.1016/S0140-6736(17)31791-9. 28716314

[15] Singh N. , Halliday A. C. , and Thomas J. M. , et al.A Safe Lithium Mimetic for Bipolar Disorder, Nature Communications. (2013) 4, no. 1, 10.1038/ncomms2320, 1332. PMC3605789 23299882

[16] Kil J. , Harruff E. E. , and Longenecker R. J. , Development of Ebselen for the Treatment of Sensorineural Hearing Loss and Tinnitus, Hearing Research. (2022) 413, 10.1016/j.heares.2021.108209, 108209. 33678494

[17] Giovanni R. , Andrea M. , and Pablo Andrei N. , et al.The Potential of Ebselen Against Bipolar Disorder: A Perspective on the Interaction With Inositol Monophosphatase (IMPase), Current Organic Chemistry. (2022) 26, no. 16, 1503–1511, 10.2174/1385272827666221130122416.

[18] Alves de Lima E. S. A. and Rio-Tinto A. , Ebselen: A Promising Repurposing Drug to Treat Infections Caused by Multidrug-Resistant Microorganisms, Interdisciplinary Perspectives on Infectious Diseases. (2024) 2024, 10.1155/2024/9109041, 9109041. 38586592 PMC10998725

[19] Sies H. and Parnham M. J. , Potential Therapeutic Use of Ebselen for COVID-19 and Other Respiratory Viral Infections, Free Radical Biology and Medicine. (2020) 156, 107–112, 10.1016/j.freeradbiomed.2020.06.032. 32598985 PMC7319625

[20] Ramli F. F. , Singh N. , and Emir U. E. , et al.Effects of Ebselen Addition on Emotional Processing and Brain Neurochemistry in Depressed Patients Unresponsive to Antidepressant Medication, Translational Psychiatry. (2024) 14, no. 1, 10.1038/s41398-024-02899-8. PMC11076504 38714646

[21] Ramli F. F. , Cowen P. J. , and Godlewska B. R. , The Potential Use of Ebselen in Treatment-Resistant Depression, Pharmaceuticals. (2022) 15, no. 4, 10.3390/ph15040485. PMC9030939 35455482

[22] Barbosa N. V. , Aschner M. , Tinkov A. A. , Farina M. , and Rocha JBTd , Should Ebselen be Considered for the Treatment of Mercury Intoxication? A Minireview, Toxicology Mechanisms and Methods. (2024) 34, no. 1, 1–12, 10.1080/15376516.2023.2258958. 37731353 PMC10841883

[23] Ghazaiean M. , Aliasgharian A. , Karami H. , and Darvishi-Khezri H. , Ebselen: A Promising Therapy Protecting Cardiomyocytes From Excess Iron in Iron-Overloaded Thalassemia Patients, Open Medicine. (2023) 18, no. 1, 10.1515/med-2023-0733, 20230733. 37465348 PMC10350894

[24] Romero-Neto I. , Dall’Anese J. , and Piano T. G. R. , et al.Repurposing Ebselen for a Potential Blending-Based Therapeutic Strategy Against Gastrointestinal Nematodes of Small Ruminants, Research in Veterinary Science. (2026) 198, 10.1016/j.rvsc.2025.105980, 105980. 41297446

[25] Allameh A. , Niayesh-Mehr R. , Aliarab A. , Sebastiani G. , and Pantopoulos K. , Oxidative Stress in Liver Pathophysiology and Disease, Antioxidants. (2023) 12, no. 9, 10.3390/antiox12091653, 1653. 37759956 PMC10525124

[26] Martín-Fernández M. , Arroyo V. , and Carnicero C. , et al.Role of Oxidative Stress and Lipid Peroxidation in the Pathophysiology of NAFLD, Antioxidants. (2022) 11, no. 11, 10.3390/antiox11112217, 2217. 36358589 PMC9686676

[27] Brzački V. , Mladenović B. , and Dimić D. , et al.Comparison Between the Effects of Selenomethionine and S-Adenosylmethionine in Preventing Cholestasis-Induced Rat Liver Damage, Amino Acids. (2019) 51, no. 5, 795–803, 10.1007/s00726-019-02716-3. 30879149

[28] Jiang Q. , Pan Y. , Cheng Y. , and Li H. , Protection of Rat Liver Against Hepatic Ischemia-Reperfusion Injury by a Novel Selenocysteine-Containing 7-Mer Peptide, Molecular Medicine Reports. (2016) 14, no. 3, 2007–2015, 10.3892/mmr.2016.5464. 27431272 PMC4991737

[29] Shang N. , Wang X. , Shu Q. , Wang H. , and Zhao L. , The Functions of Selenium and Selenoproteins Relating to the Liver Diseases, Journal of Nanoscience and Nanotechnology. (2019) 19, no. 4, 1875–1888, 10.1166/jnn.2019.16287. 30486929

[30] Reja M. , Makar M. , Visaria A. , Marino D. , and Rustgi V. , Increased Serum Selenium Levels are Associated With Reduced Risk of Advanced Liver Fibrosis and All-Cause Mortality in NAFLD Patients: National Health and Nutrition Examination Survey (NHANES) III, Annals of Hepatology. (2020) 19, no. 6, 635–640, 10.1016/j.aohep.2020.07.006. 32745632

[31] Liang Q. , Huang R. , Peng Z. , and Zou M. , Impact of Dietary Selenium and Blood Concentration on Liver Function: A Population-Based Study, Frontiers in Nutrition. (2024) 11, 10.3389/fnut.2024.1415288, 1415288. 39086539 PMC11288839

[32] Lu Q. , Cai Y. , and Xiang C. , et al.Ebselen, a Multi-Target Compound: Its Effects on Biological Processes and Diseases, Expert Reviews in Molecular Medicine. (2021) 23, 10.1017/erm.2021.14, e12.

[33] Nogueira C. W. , Barbosa N. V. , and Rocha J. B. T. , Toxicology and Pharmacology of Synthetic Organoselenium Compounds: An Update, Archives of Toxicology. (2021) 95, no. 4, 1179–1226, 10.1007/s00204-021-03003-5. 33792762 PMC8012418

[34] Meotti F. C. , Borges V. C. , Zeni G. , Rocha J. B. , and Nogueira C. W. , Potential Renal and Hepatic Toxicity of Diphenyl Diselenide, Diphenyl Ditelluride and Ebselen for Rats and Mice, Toxicology Letters. (2003) 143, no. 1, 9–16, 10.1016/S0378-4274(03)00090-0. 12697375

[35] Caeran Bueno D. , Meinerz D. F. , and Allebrandt J. , et al.Cytotoxicity and Genotoxicity Evaluation of Organochalcogens in Human Leucocytes: A Comparative Study Between Ebselen, Diphenyl Diselenide, and Diphenyl Ditelluride, BioMed Research International. (2013) 2013, 10.1155/2013/537279, 537279. 24350274 PMC3856129

[36] Sies H. , Metabolism and Disposition of Ebselen, *Proceedings of the Selenium in Biology and Medicine*, 1989, Springer, 153–162.

[37] Morin D. , Zini R. , Ligeret H. , Neckameyer W. , Labidalle S. , and Tillement J. P. , Dual Effect of Ebselen on Mitochondrial Permeability Transition, Biochemical Pharmacology. (2003) 65, no. 10, 1643–1651, 10.1016/S0006-2952(03)00114-X. 12754100

[38] Farina M. , Soares F. A. A. , Zeni G. , Souza D. O. , and Rocha J. B. T. , Additive Pro-Oxidative Effects of Methylmercury and Ebselen in Liver From Suckling Rat Pups, Toxicology Letters. (2004) 146, no. 3, 227–235, 10.1016/j.toxlet.2003.10.001. 14687760

[39] Berumen J. , Baglieri J. , Kisseleva T. , and Mekeel K. , Liver Fibrosis: Pathophysiology and Clinical Implications, WIREs Mechanisms of Disease. (2021) 13, no. 1, 10.1002/wsbm.1499, e1499. 32713091 PMC9479486

[40] Sultana M. , Islam M. A. , Khairnar R. , and Kumar S. , A Guide to Pathophysiology, Signaling Pathways, and Preclinical Models of Liver Fibrosis, Molecular and Cellular Endocrinology. (2025) 598, 10.1016/j.mce.2024.112448, 112448. 39755140

[41] Li S. , Tan H. Y. , and Wang N. , et al.The Role of Oxidative Stress and Antioxidants in Liver Diseases, International Journal of Molecular Sciences. (2015) 16, no. 11, 26087–26124, 10.3390/ijms161125942. 26540040 PMC4661801

[42] Cederbaum A. I. , Role of CYP2E1 in Ethanol-Induced Oxidant Stress, Fatty Liver and Hepatotoxicity, Digestive Diseases. (2010) 28, no. 6, 802–811, 10.1159/000324289. 21525766 PMC3211519

[43] Wasser S. , Lim G. Y. , Ong C. N. , and Tan C. E. , Anti-Oxidant Ebselen Causes the Resolution of Experimentally Induced Hepatic Fibrosis in Rats, Journal of Gastroenterology and Hepatology. (2001) 16, no. 11, 1244–1253, 10.1046/j.1440-1746.2001.02621.x. 11903743

[44] Al Aboud D. , Baty R. S. , and Alsharif K. F. , et al.Protective Efficacy of Thymoquinone or Ebselen Separately Against Arsenic-Induced Hepatotoxicity in Rat, Environmental Science and Pollution Research. (2021) 28, no. 5, 6195–6206, 10.1007/s11356-020-10955-1. 32989703

[45] Pan M. , Cai C. , and Li W. , et al.Ebselen Improves Lipid Metabolism by Activating PI3K/Akt and Inhibiting TLR4/JNK Signaling Pathway to Alleviate Nonalcoholic Fatty Liver, Cytokine. (2024) 181, 10.1016/j.cyto.2024.156671, 156671. 38943739

[46] Lu J.-L. , Yu C.-X. , and Song L.-J. , Programmed Cell Death in Hepatic Fibrosis: Current and Perspectives, Cell Death Discovery. (2023) 9, no. 1, 10.1038/s41420-023-01749-8. PMC10716404 38086792

[47] Motola-Kuba D. , Zamora-Valdés D. , Uribe M. , and Méndez-Sánchez N. , Hepatocellular Carcinoma. An overview, Annals of Hepatology. (2006) 5, no. 1, 16–24, 10.1016/S1665-2681(19)32034-4. 16531960

[48] Ye Y. , Yu B. , Wang H. , and Yi F. , Glutamine Metabolic Reprogramming in Hepatocellular Carcinoma, Frontiers in Molecular Biosciences. (2023) 10, 10.3389/fmolb.2023.1242059, 1242059. 37635935 PMC10452011

[49] Wang J. B. , Erickson J. W. , and Fuji R. , et al.Targeting Mitochondrial Glutaminase Activity Inhibits Oncogenic Transformation, Cancer Cell. (2010) 18, no. 3, 207–219, 10.1016/j.ccr.2010.08.009. 20832749 PMC3078749

[50] Thomas A. G. , Rojas C. , and Tanega C. , et al.Kinetic Characterization of Ebselen, Chelerythrine and Apomorphine as Glutaminase Inhibitors, Biochemical and Biophysical Research Communications. (2013) 438, no. 2, 243–248, 10.1016/j.bbrc.2013.06.110. 23850693 PMC4094369

[51] Shaaban S. , Negm A. , Sobh M. A. , and Wessjohann L. A. , Organoselenocyanates and Symmetrical Diselenides Redox Modulators: Design, Synthesis and Biological Evaluation, European Journal of Medicinal Chemistry. (2015) 97, 190–201, 10.1016/j.ejmech.2015.05.002. 25969171

[52] Yang C. F. , Shen H. M. , and Ong C. N. , Ebselen Induces Apoptosis in HepG(2) Cells Through Rapid Depletion of Intracellular Thiols, Archives of Biochemistry and Biophysics. (2000) 374, no. 2, 142–152, 10.1006/abbi.1999.1574. 10666292

[53] Nakamura Y. , Feng Q. , and Kumagai T. , et al.Ebselen, a Glutathione Peroxidase Mimetic Seleno-Organic Compound, as a Multifunctional Antioxidant: Implication for Inflammation-Associated Carcinogenesis ^∗^ , Journal of Biological Chemistry. (2002) 277, no. 4, 2687–2694, 10.1074/jbc.M109641200. 11714717

[54] Xiao H. and Parkin K. L. , Induction of Phase II Enzyme Activity by Various Selenium Compounds, Nutrition and Cancer-an International Journal. (2006) 55, no. 2, 210–223, 10.1207/s15327914nc5502_13. 17044777

[55] Yang C. F. , Liu J. , Wasser S. , Shen H. M. , Tan C. E. , and Ong C. N. , Inhibition of Ebselen on Aflatoxin B(1)-Induced Hepatocarcinogenesis in Fischer 344 Rats, Carcinogenesis. (2000) 21, no. 12, 2237–2243, 10.1093/carcin/21.12.2237. 11133813

[56] Patwardhan R. S. , Sharma D. , and Sandur S. K. , Thioredoxin Reductase: An Emerging Pharmacologic Target for Radiosensitization of Cancer, Translational Oncology. (2022) 17, 10.1016/j.tranon.2022.101341, 101341. 35078017 PMC8790659

[57] Lei H. , Wang G. , Zhang J. , and Han Q. , Inhibiting TrxR Suppresses Liver Cancer by Inducing Apoptosis and Eliciting Potent Antitumor Immunity, Oncology Reports. (2018) 40, 3447–3457, 10.3892/or.2018.6740. 30272318 PMC6196602

[58] Lee D. , Xu I. M. , and Chiu D. K. , et al.Induction of Oxidative Stress Through Inhibition of Thioredoxin Reductase 1 is an Effective Therapeutic Approach for Hepatocellular Carcinoma, Hepatology. (2019) 69, no. 4, 1768–1786, 10.1002/hep.30467. 30561826 PMC8690574

[59] Vasukutty A. , Bhattarai P. Y. , and Choi H. S. , Ebselen Suppresses Breast Cancer Tumorigenesis by Inhibiting YTHDF1-Mediated c-Fos Expression, International Journal of Molecular Sciences. (2025) 26, no. 19, 10.3390/ijms26199416, 9416. 41096691 PMC12524890

[60] Feng Q. , Li X. , and Sun W. , et al.Discovery of Ebselen as an Inhibitor of 6PGD for Suppressing Tumor Growth, Cancer Management and Research. (2020) 12, 6921–6934, 10.2147/CMAR.S254853. 32801914 PMC7415460

[61] Bijian K. , Zhang Z. , and Xu B. , et al.Synthesis and Biological Activity of Novel Organoselenium Derivatives Targeting Multiple Kinases and Capable of Inhibiting Cancer Progression to Metastases, European Journal of Medicinal Chemistry. (2012) 48, 143–152, 10.1016/j.ejmech.2011.12.006. 22204902

[62] Blasquez L. , Bouzinba-Segard H. , Bourdoulous S. , and Faure C. , Ebselen Oxide and Derivatives are New Allosteric HER2 Inhibitors for HER2-Positive Cancers, Molecular Oncology. (2023) 17, no. 10, 1981–1999, 10.1002/1878-0261.13419. 36912768 PMC10552892

[63] Zhong M. , Bi Y. , and Zhang J. , et al.Synthesis, Anticancer Activity and Molecular Modeling of New NSAIDs-Ebselen Derivatives as Potential TrxR1 Inhibitor, Results in Chemistry. (2025) 18, 10.1016/j.rechem.2025.102802, 102802.

[64] Yan J. , Guo Y. , Wang Y. , Mao F. , Huang L. , and Li X. , Design, Synthesis, and Biological Evaluation of Benzoselenazole-Stilbene Hybrids as Multi-Target-Directed Anti-Cancer Agents, European Journal of Medicinal Chemistry. (2015) 95, 220–229, 10.1016/j.ejmech.2015.03.030. 25817772

[65] Engman L. , Cotgreave I. , Angulo M. , Taylor C. W. , Paine-Murrieta G. D. , and Powis G. , Diaryl Chalcogenides as Selective Inhibitors of Thioredoxin Reductase and Potential Antitumor Agents, Anticancer Research. (1997) 17, no. 6D, 4599–4605. 9494575

[66] Kwak M. K. , Egner P. A. , and Dolan P. M. , et al.Role of Phase 2 Enzyme Induction in Chemoprotection by Dithiolethiones, Mutation Research. (2001) 480-481, 305–315, 10.1016/S0027-5107(01)00190-7. 11506823

[67] Lee S. B. , Kim C. Y. , Lee H. J. , Yun J. H. , and Nho C. W. , Induction of the Phase II Detoxification Enzyme NQO1 in Hepatocarcinoma Cells by Lignans From the Fruit of *Schisandra chinensis* Through Nuclear Accumulation of Nrf2, Planta Medica. (2009) 75, no. 12, 1314–1318, 10.1055/s-0029-1185685. 19452436

[68] El-Bayoumy K. , The Protective Role of Selenium on Genetic Damage and on Cancer, Mutation Research. (2001) 475, no. 1-2, 123–139, 10.1016/S0027-5107(01)00075-6. 11295158

[69] Lü J. , Jiang C. , and Hu H. , Selenium Compounds for Cancer Prevention and Therapy—Human Clinical Trial Considerations, Medical Review. (2025) 5, no. 3, 203–230, 10.1515/mr-2024-0065. 40600186 PMC12207204

[70] Kim H. , Jang M. , and Kim E. , Exploring the Multifunctional Role of Alpha-Fetoprotein in Cancer Progression: Implications for Targeted Therapy in Hepatocellular Carcinoma and Beyond, International Journal of Molecular Sciences. (2025) 26, no. 10, 10.3390/ijms26104863, 4863. 40430002 PMC12112184

[71] Nagy L. E. , McQueen C. A. , 2.11-Mechanisms of Hepatic Steatosis☆, Comprehensive Toxicology (Third Edition), 2018, Elsevier, 296–309.

[72] Hou X. , Wang Z. , and Peng M. , Selenium Compounds and Their Bioactivities: Molecular Mechanisms and Prospects for Functional Food and Therapeutic Applications, Plants. (2025) 14, no. 17, 10.3390/plants14172622, 2622. 40941787 PMC12430616

[73] Chen F. , Li Q. , Xu X. , and Wang F. , Selenium Attenuates Ethanol-Induced Hepatocellular Injury by Regulating Ferroptosis and Apoptosis, Turkish Journal of Gastroenterology. (2024) 35, no. 10, 778–786, 10.5152/tjg.2024.24159. PMC11465188 39412359

[74] Kono H. , Arteel G. E. , Rusyn I. , Sies H. , and Thurman R. G. , Ebselen Prevents Early Alcohol-Induced Liver Injury in Rats, Free Radical Biology and Medicine. (2001) 30, no. 4, 403–411, 10.1016/S0891-5849(00)00490-1. 11182296

[75] Oshita M. , Takei Y. , Kawano S. , Fusamoto H. , and Kamada T. , Protective Effect of Ebselen on Constrictive Hepatic Vasculature: Prevention of Alcohol-Induced Effects on Portal Pressure in Perfused Livers, Journal of Pharmacology and Experimental Therapeutics. (1994) 271, no. 1, 20–24, 10.1016/S0022-3565(25)22753-7. 7965715

[76] Pivetta L. A. , Pereira R. P. , and Farinon M. , et al.Ethanol Inhibits δ-Aminolevulinate Dehydratase and Glutathione Peroxidase Activities in Mice Liver: Protective Effects of Ebselen and N-Acetylcysteine, Environmental Toxicology and Pharmacology. (2006) 21, no. 3, 338–343, 10.1016/j.etap.2005.10.003. 21783677

[77] Koyanagi T. , Nakamuta M. , and Enjoji M. , et al.The Selenoorganic Compound Ebselen Suppresses Liver Injury Induced by *Propionibacterium acnes* and Lipopolysaccharide in Rats, International Journal of Molecular Medicine. (2001) 7, 321–327, 10.3892/ijmm.7.3.321. 11179515

[78] Tiegs G. , Küsters S. , Künstle G. , Hentze H. , Kiemer A. K. , and Wendel A. , Ebselen Protects Mice Against T Cell-Dependent, TNF-Mediated Apoptotic Liver Injury, Journal of Pharmacology and Experimental Therapeutics. (1998) 287, no. 3, 1098–1104, 10.1016/S0022-3565(24)37906-6. 9864298

[79] Wang J. F. , Komarov P. , Sies H. , and de Groot H. , Inhibition of Superoxide and Nitric Oxide Release and Protection From Reoxygenation Injury by Ebselen in Rat Kupffer Cells, Hepatology. (1992) 15, no. 6, 1112–1116, 10.1002/hep.1840150623. 1375578

[80] Wendel A. and Tiegs G. , A Novel Biologically Active Seleno-Organic Compound--VI. Protection by Ebselen (PZ 51) Against Galactosamine/Endotoxin-Induced Hepatitis in Mice, Biochemical Pharmacology. (1986) 35, no. 13, 2115–2118, 10.1016/0006-2952(86)90578-2. 3729968

[81] Konz K. H. , Tiegs G. , and Wendel A. , Protection by Ebselen Against Endotoxin Shock in Rats or Mice Sensitized by Galactosamine, Uro-Oncology: Current Status and Future Trends. (1987) 236A, 281–288. 3615436

[82] Harman A. W. , Adamson G. M. , and Shaw S. G. , Protection From Oxidative Damage in Mouse Liver Cells, Toxicology Letters. (1992) 64-65, 581–587, 10.1016/0378-4274(92)90235-C. 1471211

[83] Qiu-Ju L. , Bessems J. G. M. , Commandeur J. N. M. , Adams B. , and Vermeulen N. P. E. , Mechanism of Protection of Ebselen Against Paracetamol-Induced Toxicity in Rat Hepatocytes, Biochemical Pharmacology. (1994) 48, no. 8, 1631–1640, 10.1016/0006-2952(94)90208-9. 7980628

[84] Rocha J. B. , Gabriel D. , and Zeni G. , et al.Ebselen and Diphenyl Diselenide Change Biochemical Hepatic Responses to Overdosage With Paracetamol, Environmental Toxicology and Pharmacology. (2005) 19, no. 2, 255–261, 10.1016/j.etap.2004.07.006. 21783484

[85] Perez M. J. and Briz O. , Bile-Acid-Induced Cell Injury and Protection, World Journal of Gastroenterology. (2009) 15, no. 14, 1677–1689, 10.3748/wjg.15.1677. 19360911 PMC2668773

[86] Sousa T. , Castro R. E. , and Pinto S. N. , et al.Deoxycholic Acid Modulates Cell Death Signaling Through Changes in Mitochondrial Membrane Properties, Journal of Lipid Research. (2015) 56, no. 11, 2158–2171, 10.1194/jlr.M062653. 26351365 PMC4617403

[87] Tanaka M. , Takezawa N. , and Kumai T. , et al.Ebselen Protects Against the Reduction in Levels of Drug-Metabolizing Enzymes in Livers of Rats With Deoxycholic Acid-Induced Liver Injury, Pharmacology & Toxicology. (2002) 91, no. 2, 64–70, 10.1034/j.1600-0773.2002.910204.x. 12420794

[88] Zhao H. , Lu H. G. , Shi Y. B. , Zhao L. M. , Bai C. , and Wang X. , Role of Enteral Nutrition Supplemented With Ebselen and EHEC in Pancreatitis-Associated Multiple Organ Dysfunction in Rats, Inflammation Research. (2006) 55, no. 10, 423–429, 10.1007/s00011-006-6008-z. 17109069

[89] Zou W. , Roth R. A. , Younis H. S. , Burgoon L. D. , and Ganey P. E. , Oxidative Stress Is Important in the Pathogenesis of Liver Injury Induced by Sulindac and Lipopolysaccharide Cotreatment, Toxicology. (2010) 272, no. 1–3, 32–38, 10.1016/j.tox.2010.03.015. 20371263 PMC2898510

[90] Shimohashi N. , Nakamuta M. , and Uchimura K. , et al.Selenoorganic Compound, Ebselen, Inhibits Nitric Oxide and Tumor Necrosis Factor-Alpha Production by the Modulation of Jun-N-Terminal Kinase and the NF-Kappab Signaling Pathway in Rat Kupffer Cells, Journal of Cellular Biochemistry. (2000) 78, no. 4, 595–606, 10.1002/1097-4644(20000915)78:4<595::AID-JCB9>3.0.CO;2-B. 10861857

[91] Yerushalmi B. , Dahl R. , Devereaux M. W. , Gumpricht E. , and Sokol R. J. , Bile Acid-Induced Rat Hepatocyte Apoptosis is Inhibited by Antioxidants and Blockers of the Mitochondrial Permeability Transition, Hepatology. (2001) 33, no. 3, 616–626, 10.1053/jhep.2001.22702. 11230742

[92] Lin T. T. , Wang B. M. , and Li X. Y. , et al.An Insight Into the Protection of Rat Liver Against Ischemia/Reperfusion Injury by 2-Selenium-Bridged Beta-Cyclodextrin, Hepatology Research. (2009) 39, no. 11, 1125–1136, 10.1111/j.1872-034X.2009.00545.x. 19624763

[93] Pritsos C. A. , Sokoloff M. , and Gustafson D. L. , PZ-51 (Ebselen) in Vivo Protection Against Adriamycin-Induced Mouse Cardiac and Hepatic Lipid Peroxidation and Toxicity, Biochemical Pharmacology. (1992) 44, no. 4, 839–841, 10.1016/0006-2952(92)90427-K. 1510734

[94] Ozaki M. , Nakamura M. , Teraoka S. , and Ota K. , Ebselen, a Novel Anti-Oxidant Compound, Protects the Rat Liver From Ischemia-Reperfusion Injury, Transplant International. (1997) 10, no. 2, 96–102, 10.1111/j.1432-2277.1997.tb00548.x. 9089992

[95] Jaromin A. , Zarnowski R. , Piętka-Ottlik M. , Andes D. R. , and Gubernator J. , Topical Delivery of Ebselen Encapsulated in Biopolymeric Nanocapsules: Drug Repurposing Enhanced Antifungal Activity, Nanomedicine. (2018) 13, no. 10, 1139–1155, 10.2217/nnm-2017-0337. 29873597 PMC10334256

[96] Pi F. , Yang F. , and Zhan Z. , et al.Oral Delivery of Ebselen Nanoparticle Modulating Macrophage Polarization for Ulcerative Colitis Treatment, Cell Biomaterials. (2026) 10.1016/j.celbio.2026.100418, 100418.

[97] Yu W. , Liu T. , and Yuan Z. , et al.Polymer-Engineered Liposome-Delivered Ebselen Against Tumor Through GSH/H_2_O_2_-Responsive Disruption of Redox Homeostasis and Direct p53 Activation, Biomaterials. (2026) 326, 10.1016/j.biomaterials.2025.123698, 123698. 40992159

